# A highly prevalent SINE mutation in the myostatin (*MSTN*) gene promoter is associated with low circulating myostatin concentration in Thoroughbred racehorses

**DOI:** 10.1038/s41598-021-86783-1

**Published:** 2021-04-12

**Authors:** Victoria O’Hara, Amélie Cowan, Dominique Riddell, Claire Massey, John Martin, Richard J. Piercy

**Affiliations:** 1 Comparative Neuromuscular Diseases Laboratory, Department of Veterinary Clinical Sciences, Royal Veterinary College, Royal College Street, London, UK; 2 Johnston Racing, Kingsley Park, Middleham, Leyburn UK

**Keywords:** Genetics, Physiology

## Abstract

Horse racing is a popular and financially important industry worldwide and researchers and horse owners are interested in genetic and training influences that maximise athletic performance. An association has been found between the presence of a short interspersed nuclear element (SINE) mutation in the myostatin (*MSTN*) gene promoter and optimal race distance in Thoroughbred horses. There is previous laboratory evidence that this mutation reduces *MSTN* expression in a cell culture model and influences skeletal muscle fibre type proportions in horses. Manipulating *MSTN* expression has been proposed for illicit gene doping in human and equine athletes and already, researchers have generated homozygous and heterozygous *MSTN*-null horse embryos following CRISPR/Cas9 editing at the equine *MSTN* locus and nuclear transfer, aiming artificially to enhance performance. To date however, the role of the naturally-occurring equine *MSTN* SINE mutation in vivo has remained unclear; here we hypothesised that it reduces, but does not ablate circulating myostatin expression. Following validation of an ELISA for detection of myostatin in equine serum and using residual whole blood and serum samples from 176 Thoroughbred racehorses under identical management, horses were genotyped for the SINE mutation by PCR and their serum myostatin concentrations measured. In our population, the proportions of SINE homozygotes, heterozygotes and normal horses were 27%, 46% and 27% respectively. Results indicated that horses that are homozygous for the SINE mutation have detectable, but significantly lower (p < 0.0001) serum myostatin concentrations (226.8 pg/ml; 69.3–895.4 pg/ml; median; minimum–maximum) than heterozygous (766 pg/ml; 64.6–1182 pg/ml) and normal horses (1099 pg/ml; 187.8–1743 pg/ml). Heterozygotes have significantly lower (p < 0.0001) myostatin concentrations than normal horses. Variation in serum myostatin concentrations across horses was not influenced by age or sex. This is the first study to reveal the direct functional effect of a highly prevalent mutation in the equine *MSTN* gene associated with exercise performance. Determining the reason for variation in expression of myostatin within SINE-genotyped groups might identify additional performance-associated environmental or genetic influences in Thoroughbreds. Understanding the mechanism by which altered myostatin expression influences skeletal muscle fibre type remains to be determined.

## Introduction

Myostatin, or growth differentiation factor 8 (GDF8), is a skeletal muscle-specific paracrine hormone with an important role in muscle development^1^: it inhibits muscle hypertrophy by regulating proliferation and differentiation of myocytes^2^. Mutations in the myostatin (*MSTN*) gene that decrease the expression of myostatin induce pre-natal muscle fibre hyperplasia and post-natal hypertrophy^3^, though not the strength of individual muscle fibres^4^. Null mutations in the myostatin gene have been recorded in several species, including cattle, pigs, sheep, dogs and humans^5^. Manipulation of *MSTN* expression has been proposed as being an attractive option for illicit human and equine gene doping in sport^6,7^ and recently, researchers have generated homozygous and heterozygous *MSTN*-null horse embryos following CRISPR/Cas9 editing at the *MSTN* locus and nuclear transfer, aiming artificially to enhance equine athletic performance^8^.

**Figure 1:**
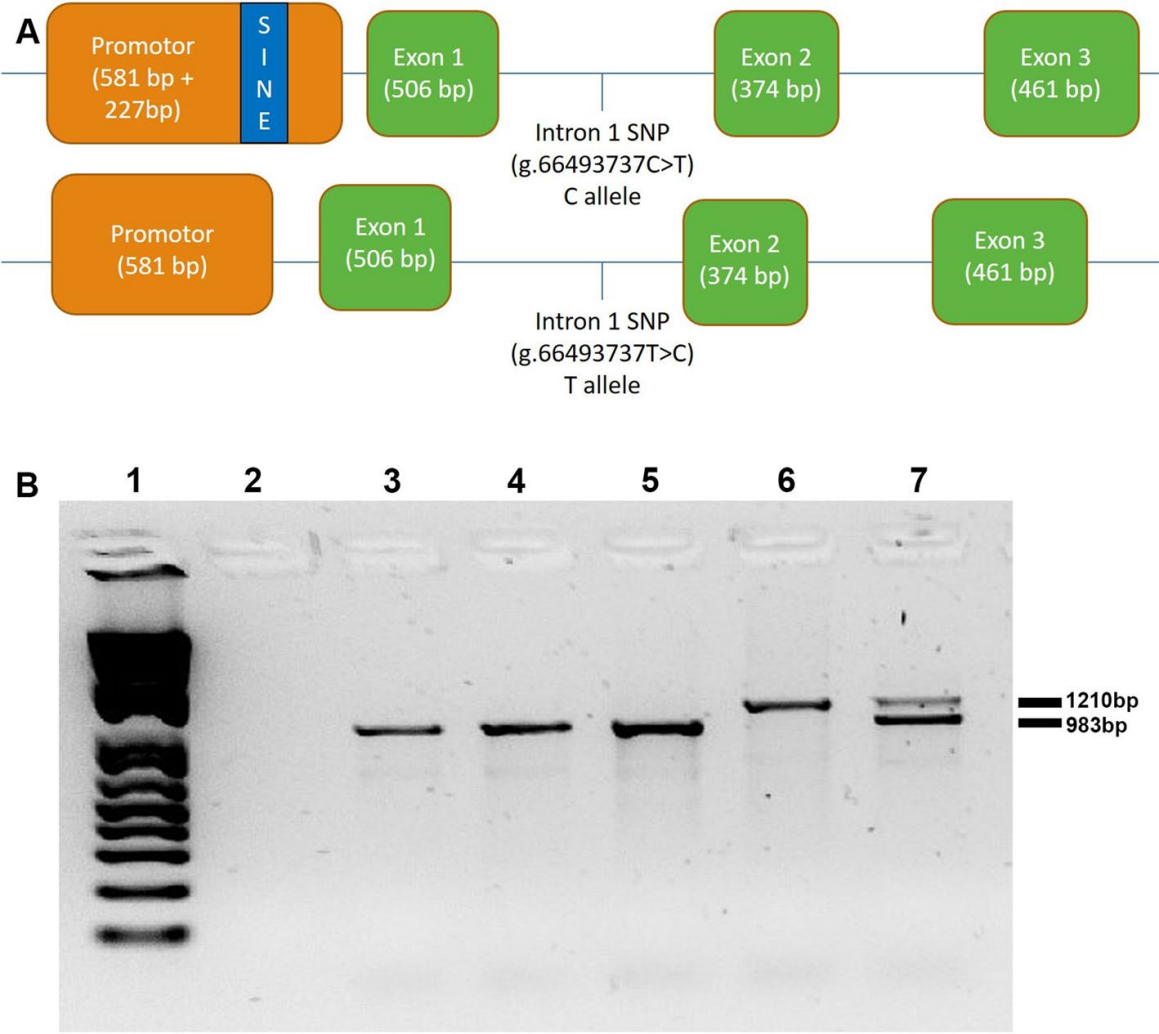
(**A**) Equine MSTN gene structure in Thoroughbred horses. (1) Note the presence of the SINE mutation within the promoter which is in linkage disequilibrium with an intronic SNP (C allele) in Thoroughbred racehorses. (2) In contrast, wild type horses that lack the SINE, have a T allele at the SNP. The equine *MSTN* gene consists of three coding exons (in green). (**B**) Genotyping agarose gel electrophoresis of PCR products designed to test for the presence or absence of the *MSTN* promoter SINE mutation. Lane 1 is a 1 kb DNA ladder followed by a water control (no band). DNA products in the following five lanes represent 3 Wild Type animals in lane 3, 4 and 5 (983 base pair (bp) product), one homozygote in lane 6 (1210 bp) and one heterozygote with both bands in lane 7 (983 bp and 1210 bp).

In horses, the *MSTN* genotype is the most important genetic contributor to a horse’s optimum race distance^4^ and it is the highest selected gene in racing Quarter Horses^9^. There are two common *MSTN* variants in Thoroughbred racehorses (Fig. 1a). The first noted was a single nucleotide polymorphism (SNP) in intron 1 associated with best race distance^10^. Horses with C:C paired alleles succeed at short races; in contrast, T:T types are better at longer, endurance races, and C:T excel at middle distances^10^. There is some debate over the origin of the C gene in the Thoroughbred, with one publication dating it to a single British mare 300 years ago^11^. In the racing industry, testing for a SNP is advocated for commercial breeding and training purposes^12^. This SNP appears to have been selected for in other equestrian disciplines, with a high representation of the T allele in Warmbloods and event horses^13,14^.

The other Thoroughbred *MSTN* variant is a SINE mutation in the gene’s promotor. Unlike the C allele, which can be found in the majority of horse breeds, this SINE is only common to Thoroughbreds and Quarter Horses^15,16^. There is strong linkage disequilibrium between the SNP and the SINE, with C:C horses having the SINE insertion and T:T horses lacking it^17^. Further, work has suggested the SINE to be the putative functional mutation rather than the intronic SNP, since reduced myostatin expression was detected in transgenic reporter assays in cultured cells driven by the promoter with and without the SINE^17,18^. The authors of a previous study concluded that the SINE moved the gene’s transcriptional start site and decreased in vitro expression of myostatin 4.5-fold^17^.

Myostatin mutations have also been selected for in other species, particularly within the meat industry, evident in ‘double muscled’ beef cattle breeds such as the Belgian Blue. These animals have a deletion (frame-shift) mutation that prevents the expression of myostatin, as in some lines of knockout mice^19,20^. Myostatin knockout animals have significantly higher proportions of fast glycolytic type 2 muscle fibres than controls^21^. Similar effects are evident in horses with the SINE insertion: these animals have 12.5% more type 2X muscle fibres and fewer type 1 than those without. Type 2X fibres are fast contracting, but quicker to fatigue, compared to type 1 fibres that contract slower but have a higher oxidative capacity and thus are more suited to endurance. This observation of muscle histological characteristics in horses corroborates the identification of the performance related associations between the CC alleles, and SINE, in optimal race distance^9^. However, horses with *MSTN* mutations are exceptionally athletic, which is not seen in other species that are *MSTN* knockouts: in Whippet racing, homozygous *MSTN* null Whippets are far inferior to their heterozygous counterparts; indeed, the latter animals are significantly over represented in top races and specifically bred^22^.

To date, the functional effect of the equine *MSTN* SINE on myostatin protein expression in vivo has not been elucidated. In this work, we optimised and validated an ELISA for detection of equine myostatin in serum, and then investigated the hypothesis that the *MSTN* SINE is associated with reduced circulating myostatin protein expression in racehorses.

## Materials and methods

### Ethical approval

Ethical approval for this project was granted by the Clinical Research and Ethical Review Board of the Royal Veterinary College. Reference: URN 2018 1830-2. All methods were performed in accordance with the relevant guidelines and regulations for animal use.

### Sample collection

Residual whole EDTA blood and serum samples were obtained from 176 racing Thoroughbreds from a single yard in England (with the owners’ permission) in Spring 2019. Both serum and EDTA blood samples were frozen within 16 h of collection and stored at − 20 °C until used. Sex, age and height of each horse was recorded. There were samples from 48 mares, 25 geldings and 103 stallions.

### Quantifying serum myostatin

Serum myostatin concentrations were quantified (with the researcher blinded to the *MSTN* genotype), by ELISA (GDF-8/Myostatin ELISA: R&D systems) with optical density recorded by plate reader at a wavelength of 450 nm with a reference of 570 nm to correct for imperfections in the plate, according to the manufacturer’s instructions. Initially, a serial dilution was performed to ascertain linearity. To examine the influence of storage on myostatin stability, myostatin concentrations were assayed in five serum samples from different horses, and then compared with the same samples that had been frozen at − 80 °C for 30 min, then thawed at room temperature on six occasions, and also in samples that were left at room temperature for 24 h before testing. Thereafter, myostatin was measured by ELISA in all 176 horse serum samples in duplicate. Controls of sterile water and buffer solution were used for every 14 samples.

### Genotyping

DNA extraction was performed on frozen EDTA blood samples using a commercial kit (Illustra Nucleon BACC 3 Genomic DNA extraction kit, G E Healthcare), as per manufacturer’s instructions. Concentration and purity were measured (NanoDrop One; ThermoFisher Scientific) and PCR was used to determine the presence of the 227 base pair SINE insertion in the *MSTN* promotor, using GoTaq Hot start polymerase (Promega) and associated reagents and the following primers: 5′–CTG ACA TTA TGC CCT GGT AA–3′ (Forward), 5′–CGC TGT TCT CAT TTA GAT CC–3′ (Reverse). Assays were run in the following conditions: 95 °C for 5 min followed by 35 cycles of 95 °C for 15 s, 55 °C for 15 s, 72 °C for 90 s, and finally 72 °C for 7 min. DNA-free negative controls (sterile water) were included.

Electrophoresis was conducted in 1.5% agarose gels. Presence of the SINE mutation was indicated by a band size of 1210 bp, and absence, by a band size of 983 bp. Heterozygous horses had bands at both 1210 bp and 983 bp (Fig. 1b). In this study, homozygous refers to animals with two SINE mutations, and heterozygous with one. Wild type (WT)/normal applies to animals that do not have the mutation.

### Statistical analysis

Statistical analysis was performed using GraphPad Prism v. 8.1.2 with the mean of the technical duplicate myostatin concentration for each horse used. For all ELISA results, a second order polynomial (quadratic) interpolated curve was used to ascertain the concentration of myostatin present in samples compared to a standard serial dilution curve, completed with each plate. For the serial dilution series Pearson’s correlation was used. Normality of distribution of bench and freeze–thaw samples, and concentrations within genotyped and signalment groups were examined visually, and by D'Agostino–Pearson normality test. Data was not normally distributed. Consequently, a non-parametric one-way repeated measure Friedmann test was used to compare effect of sample storage condition with each sample normalised to the value of its fresh serum myostatin concentration. A Kruskal–Wallis test was used to compare serum myostatin concentration between genotyped and signalment groups with post hoc multiple comparison testing performed using a Tukey test. The sex distribution within genotyped groups was compared by Chi squared test. Any association between horse height and serum myostatin concentration was examined by Pearson correlation. In all cases, differences were considered statistically significantly different when P < 0.05.

## Results

### Initial validation

Serial dilution revealed both linearity and accuracy (R^2^ = 0.999, P < 0.0001) and enabled selection of an appropriate serum dilution for ongoing tests. Serum was diluted at 1:10 to allow for variations between horses whilst remaining within the linear range. Myostatin concentration was measured in identical sourced samples of fresh serum, frozen and thawed, and serum left at room temperature for 24 h (n = 5). No significant differences (P = 0.37) were detected within samples stored in different ways suggesting that equine myostatin is stable in serum at room temperature for 24 h and can withstand repeated freeze–thaw cycles (results not shown).

### Influence of horse *MSTN* genotype on serum myostatin concentration

**Figure 2:**
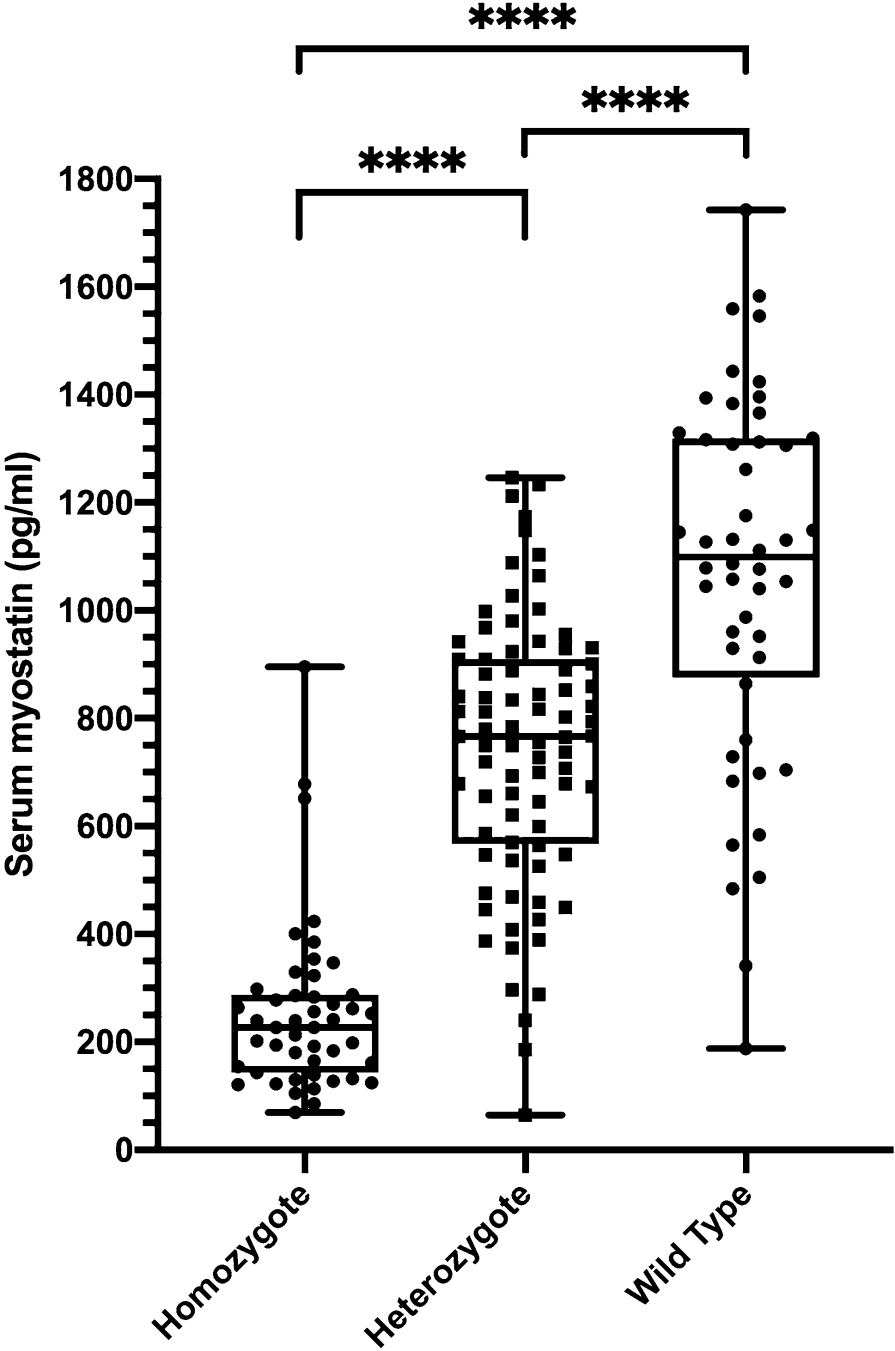
Comparison of serum myostatin concentrations between Thoroughbred horses of different *MSTN* genotypes. Note that homozygotes (n = 47) have significantly lower (but still detectable) serum myostatin concentrations than heterozygotes (n = 81) which in turn have lower concentrations than wild type horses (n = 48). There is substantial overlap in serum myostatin concentrations, particularly between heterozygotes and wild type horses. Individual data points shown and boxes represent the median and interquartile ranges and whiskers the range. (****P < 0.0001).

Homozygotes (n = 47) had significantly lower (though detectable) serum myostatin concentrations than heterozygous horses (n = 81) (P < 0.0001) which in turn had significantly lower myostatin concentrations than wild type horses (n = 48) (P < 0.0001). There was prominent overlap of the range of myostatin concentrations in heterozygotes and wild type horses (Fig. 2). Within each group, occasional outliers were identified.

**Figure 3:**
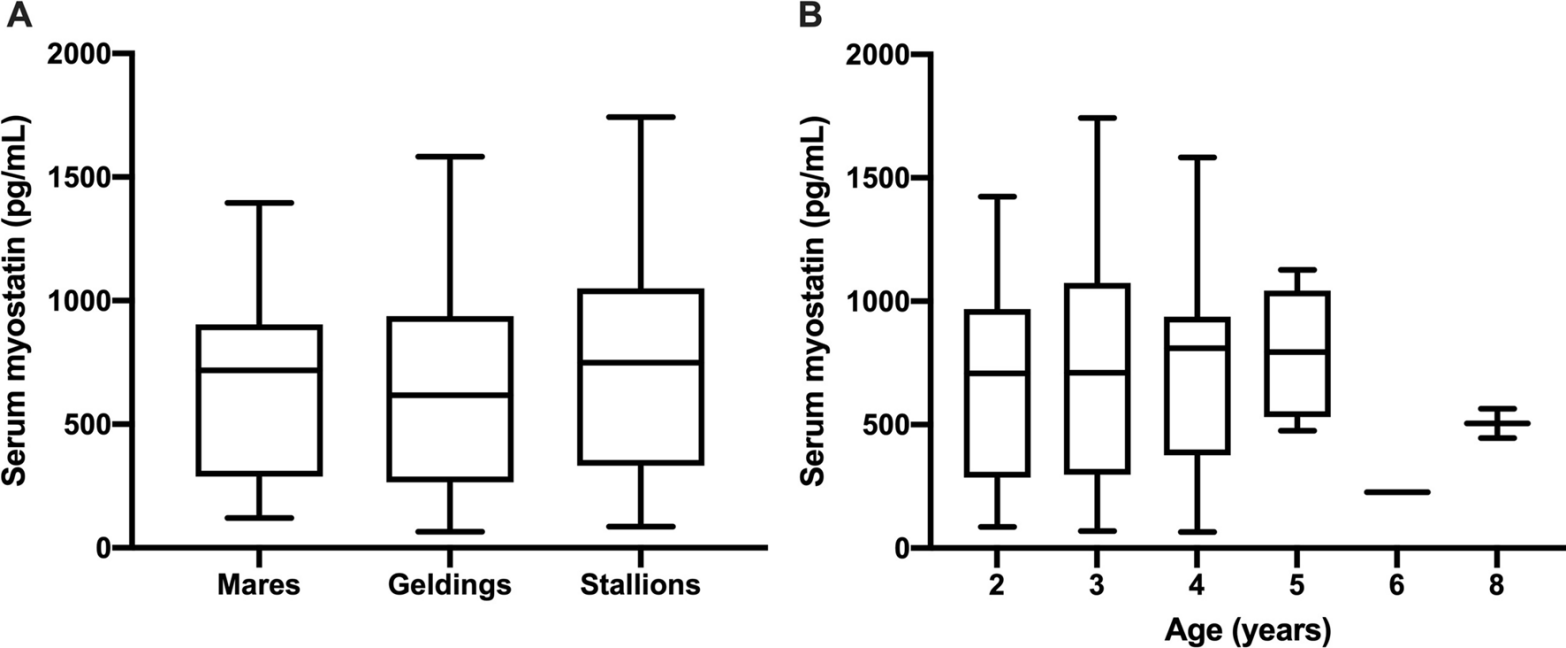
Comparison of serum myostatin concentrations in horses of different sex (**A**) and age (**B**). Boxes represent the median and interquartile ranges and whiskers the range. No significant differences detected (P = 0.14 and 0.72 respectively).

To further explore possible reasons for variations in serum myostatin concentration, the effect of sex and age were considered. There was no significant difference in the proportions of sexes (mare, gelding, stallion) in each genotyped group (P = 0.25) and no difference in median ages between genotyped groups (P = 0.33) (data not shown). Further, there were no significant differences in serum myostatin concentrations between horses of different sexes (Fig. 3A) or between horses of different ages across all groups (Fig. 3B).

Given that myostatin has prominent effects on musculature and that muscles and the skeleton are directly associated, we speculated that serum myostatin concentration might be associated with horse height. No significant associations were found between the two variables in homozygotes (P = 0.32) and WT horses (P = 0.76), but there was a significant, though very weak association between the two in heterozygotes (P = 0.003; R^2^ = 0.10).

## Discussion

Previously, work has identified genetic predictors of exercise performance or racing success, but the functional effect of these variants has been less well explored. In this study we sought to test the use of a commercial ELISA for detection of myostatin in equine serum and thereafter to examine the effects of a *MSTN* gene promoter SINE insertion in vivo. Crucially, this study has revealed the biological consequences of the SINE mutation at the protein level in Thoroughbred racehorses. The results corroborate those found in previous in vitro cell culture reporter studies^17,18^ and help explain in vivo effects on performance.

Morrison et al*.* described use of an ELISA for detection of serum myostatin in mixed breed horses and ponies of unknown *MSTN* genotype revealing that circulating myostatin concentration is significantly higher in obese compared to lean animals^23^. All the Thoroughbreds used in the current trial were all from the same racing yard, and were of similar lean body condition; however, given the variation of myostatin expression even within genotyped groups, it would be of interest to see if body condition score is also associated with myostatin serum concentration in racehorses.

There is debate as to whether sex affects serum myostatin concentration in humans. Tanaka et al*.* revealed that obese men had higher circulating myostatin concentration than obese women^24^. Similarly, it has also been suggested that only men have an association between muscle mass and serum myostatin^25^. However, Yano et al*.* found no difference in serum myostatin between sexes^26^, as we report in the current work. Circulating myostatin concentrations decline with age in mammals, including horses^27^ and there is much interest in the hormone’s role in development of age-associated sarcopaenia in older mammals^28^. An association with age was not seen in this study; however, the majority of horses in this investigation were between 2 and 3 years old (i.e. active racehorses), with the oldest being 8. As such, serum myostatin concentrations might decline in older horses than those in this study.

Physically fit humans have higher serum myostatin concentrations than unfit humans^29^. In the current work all samples were collected from racehorses in training, and likely therefore with similar levels of fitness. However, other work has found that musculoskeletal condition has little effect on serum myostatin concentrations in humans^30–32^ including those in high-velocity resistance training^33^. Recent studies suggest that myostatin expression changes in equine skeletal muscle in response to exercise and training^34^: in future it would be of interest to examine the effects of exercise and training on serum myostatin concentrations within *MSTN*-genotyped horse groups.

*MSTN* genotype is associated with variation in muscle mass^35^: WT horses have the lowest muscle mass, and those with SINE mutations have the highest. Similarly, a study in a child with marked muscle hypertrophy detected exceedingly low myostatin concentrations in the individual’s serum^36^. This is comparable to the effect seen in homozygous *MSTN*-null, double-muscled meat-producing animals that have significantly lower circulating myostatin than wild type counterparts. Homozygous SINE horses also have a greater body weight to wither height ratio^35^. We found no difference in the height of homozygous and wild type horses but since horses were not weighed, the mass to height ratio could not be calculated; our data does though suggest that the SINE mutation in horses is not directly linked to stature. Myostatin has an indirect effect on bone formation due to decreased muscle growth and thereby a reduction in mechanical loading. The protein is also expressed around fracture sites and reduces callus formation^37–39^. Wu et al*.* discovered that circulating myostatin correlates with lower bone density^30^. Consequently, it would be of interest to examine whether horses with the SINE insertion are more at risk of musculoskeletal injury.

In this study, myostatin was detectable in the serum of homozygous SINE animals. Unlike Belgian Blue cattle^19^, mice^20^ and dogs^22^ with null mutations that prevent the expression of myostatin entirely, these horses produce the hormone, albeit at a lower concentration than the heterozygous and WT animals. This may be the reason that these animals are outstanding athletes whereas those with myostatin mutations in other species are not. Similarly, perhaps, heterozygous “bully” (myostatin haploinsufficient) Whippets are faster than wild type dogs^22^ and notably, the mother of a human myostatin null infant (who was therefore presumably a heterozygote) was an Olympic athlete^36^.

Whilst the range of myostatin concentrations in homozygous horses is clearly defined, there is a marked overlap between heterozygous and WT horses. It seems likely therefore that there are other genetic or environmental factors that influence serum myostatin concentration in these animals. It will be of interest to examine daily and seasonal variability within horses and to determine whether heterozygote horses that have consistently lower serum myostatin concentrations excel over shorter distances, similar to homozygous types. Determining the reasons for this variation might reveal other performance-associated gene variants in Thoroughbreds.

To conclude, within Thoroughbreds the circulating myostatin concentration differs, and is dependent on the number of copies of a SINE mutation within the *MSTN* gene promoter. Determining the reason why myostatin expression varies between horses of the same genotype might reveal additional performance-associated genes. Finally, the underlying mechanism that links altered myostatin expression with the fibre type proportions of skeletal muscles in mammals remains unknown and is worthy of further study.

## Abbreviations

ELISA: Enzyme Linked Immunosorbent Assay
GDF8: Growth differentiation factor 8
MSTN: Myostatin
SINE: Short interspersed nuclear element
SNP: Single nucleotide polymorphism

## References

[1] Beyer TA, Narimatsu M, Weiss A, David L, Wrana JL (2013). The TGFβ superfamily in stem cell biology and early mammalian embryonic development. Biochimica et Biophysica Acta (BBA) General Subjects.

[2] Trendelenburg AU (2009). Myostatin reduces Akt/TORC1/p70S6K signaling, inhibiting myoblast differentiation and myotube size. Am. J. Physiol. Cell Physiol..

[3] Matsakas A, Otto A, Elashry MI, Brown SC, Patel K (2010). Altered primary and secondary myogenesis in the myostatin-null mouse. Rejuvenation Res..

[4] Hill EW (2019). The contribution of myostatin (MSTN) and additional modifying genetic loci to race distance aptitude in Thoroughbred horses racing in different geographic regions. Equine Vet. J..

[5] Aiello DPK, Lasagna E (2018). The myostatin gene: An overview of mechanisms of action and its relevance to livestock animals. Anim. Genet..

[6] Brzezianska E, Domanska D, Jegier A (2014). Gene doping in sport—Perspectives and risks. Biol. Sport.

[7] Tozaki T (2020). Whole-genome resequencing using genomic DNA extracted from horsehair roots for gene-doping control in horse sports. J. Equine Sci..

[8] Moro LN (2020). Generation of myostatin edited horse embryos using CRISPR/Cas9 technology and somatic cell nuclear transfer. Sci. Rep..

[9] Petersen JL (2013). Genome-wide analysis reveals selection for important traits in domestic horse breeds. PLoS Genet..

[10] Hill EW (2010). A sequence polymorphism in MSTN predicts sprinting ability and racing stamina in thoroughbred horses. PLoS ONE.

[11] Bower MA (2012). The genetic origin and history of speed in the Thoroughbred racehorse. Nat. Commun..

[12] Hill EW, Ryan DP, MacHugh DE (2012). Horses for courses: A DNA-based test for race distance aptitude in thoroughbred racehorses. Recent Pat. DNA Gene Seq..

[13] Serpa PB, Garbade P, Natalini CC, Pires AR, Tisotti TM (2017). High-resolution melting analysis for detection of a single-nucleotide polymorphism and the genotype of the myostatin gene in warmblood horses. Am. J. Vet. Res..

[14] Padilha FGF, El-Jaick KB, de Castro L, Moreira ADS, Ferreira AMR (2018). Effect of selection for eventing on the MSTN gene in Brazilian sport horses. J. Equine Sci..

[15] Dall'Olio S, Scotti E, Fontanesi L, Tassinari M (2014). Analysis of the 227 bp short interspersed nuclear element (SINE) insertion of the promoter of the myostatin (MSTN) gene in different horse breeds. Vet. Ital..

[16] Petersen JL, Valberg SJ, Mickelson JR, McCue ME (2014). Haplotype diversity in the equine myostatin gene with focus on variants associated with race distance propensity and muscle fiber type proportions. Anim. Genet..

[17] Rooney MF, Hill EW, Kelly VP, Porter RK (2018). The, “speed gene” effect of myostatin arises in Thoroughbred horses due to a promoter proximal SINE insertion. PLoS ONE.

[18] Santagostino M (2015). Genome-wide evolutionary and functional analysis of the Equine Repetitive Element 1: An insertion in the myostatin promoter affects gene expression. BMC Genet..

[19] Kambadur R, Sharma M, Smith TP, Bass JJ (1997). Mutations in myostatin (GDF8) in double-muscled Belgian Blue and Piedmontese cattle. Genome Res..

[20] McPherron AC, Lee S-J (1997). Double muscling in cattle due to mutations in the myostatin gene. Proc. Natl. Acad. Sci..

[21] Deveaux V, Cassar-Malek I, Picard B (2001). Comparison of contractile characteristics of muscle from Holstein and double-muscled Belgian Blue foetuses. Comp. Biochem. Physiol. A Mol. Integr. Physiol..

[22] Mosher DS (2007). A mutation in the myostatin gene increases muscle mass and enhances racing performance in heterozygote dogs. PLoS Genet..

[23] Morrison PK (2014). Preliminary investigation into a potential role for myostatin and its receptor (ActRIIB) in lean and obese horses and ponies. PLoS ONE.

[24] Tanaka M (2018). Role of serum myostatin in the association between hyperinsulinemia and muscle atrophy in Japanese obese patients. Diabetes Res. Clin. Pract..

[25] Peng L-N, Lee W-J, Liu L-K, Lin M-H, Chen L-K (2018). Healthy community-living older men differ from women in associations between myostatin levels and skeletal muscle mass. J. Cachexia Sarcopenia Muscle.

[26] Yano S (2015). Relationship between blood myostatin levels and kidney function: Shimane CoHRE Study. PLoS ONE.

[27] Poggioli T (2016). Circulating growth differentiation factor 11/8 levels decline with age. Circ. Res..

[28] White TA, LeBrasseur NK (2014). Myostatin and sarcopenia: Opportunities and challenges—A mini-review. Gerontology.

[29] Arrieta H (2019). Serum myostatin levels are higher in fitter, more active, and non-frail long-term nursing home residents and increase after a physical exercise intervention. Gerontology.

[30] Wu L-F (2018). Relative abundance of mature myostatin rather than total myostatin is negatively associated with bone mineral density in Chinese. J. Cell Mol. Med..

[31] Koyun D, Nergizoglu G, Kir KM (2018). Evaluation of the relationship between muscle mass and serum myostatin levels in chronic hemodialysis patients. Saudi J. Kidney Dis. Transpl..

[32] Ma YLX, Zhang H, Ou Y, Zhang Z, Li S, Wu F, Sheng Z, Liao E (2016). Serum myostatin in central south Chinese postmenopausal women: Relationship with body composition, lipids and bone mineral density. Endocr. Res..

[33] Binns A, Gray M, Henson AC, Fort IL (2017). Changes in lean mass and serum myostatin with habitual protein intake and high-velocity resistance training. J. Nutr. Health Aging.

[34] Farries G (2019). Analysis of genetic variation contributing to measured speed in Thoroughbreds identifies genomic regions involved in the transcriptional response to exercise. Anim. Genet..

[35] Tozaki T (2011). Sequence variants at the myostatin gene locus influence the body composition of Thoroughbred horses. J. Vet. Med. Sci..

[36] Schuelke M (2004). Myostatin mutation associated with gross muscle hypertrophy in a child. N. Engl. J. Med..

[37] Kawao N, Kaji H (2015). Interactions between muscle tissues and bone metabolism. J. Cell. Biochem..

[38] Dankbar B (2015). Myostatin is a direct regulator of osteoclast differentiation and its inhibition reduces inflammatory joint destruction in mice. Nat. Med..

[39] Tarantino U (2015). Sarcopenia: A histological and immunohistochemical study on age-related muscle impairment. Aging Clin. Exp. Res..

